# Adaptive management in disaster crisis: Role analysis in cross-sector collaboration

**DOI:** 10.4102/jamba.v17i1.1830

**Published:** 2025-09-05

**Authors:** Intan F. Meutia, Anna G. Zainal, Bayu Sujadmiko, Muhammad S. Assalam, Rizki A. Putri

**Affiliations:** 1 Department of Public Administration, Faculty of Social and Political Sciences, University of Lampung, Bandarlampung, Indonesia; 2 Department of Communication Science, Faculty of Social and Political Sciences, University of Lampung, Bandarlampung, Indonesia; 3 Department of International Law, Faculty of Law, University of Lampung, Bandarlampung, Indonesia

**Keywords:** adaptive governance, community empowerment, disaster management, early warning, disaster management

## Abstract

Effective disaster management requires cross-actor collaboration within adaptive governance. This study examines the role of the Meteorology, Climatology and Geophysics Agency in implementing disaster governance in Lampung Province. The collaboration with other agencies remains limited and passive. Existing coordination tends to be internal, lacking clear standard procedures. Although the agency has made efforts to build social capital through digital communication and direct interaction, community involvement in decision-making is still minimal. Community empowerment and participatory communication mechanisms are key to enhancing disaster response. Furthermore, the development of employee capacity through data-driven training and partnerships with other institutions demonstrates its commitment to strengthening adaptive governance.

**Contribution:**

This study provides a comprehensive understanding of adaptive governance in disaster management by identifying specific gaps in collaboration and suggesting practical, actionable solutions for improving inter-agency coordination and community engagement.

## Introduction

The Class I Meteorological Station Radin Inten II is a public service organisation that serves as one of the Technical Implementation Units (Unit Pelaksanan Teknis [UPT]) under the Meteorology, Climatology and Geophysics Agency, located in Lampung Province. The Class I Meteorological Station Radin Inten II operates under the responsibility of the Head of the Agency and is administratively overseen by the Secretary-General, while technically and operationally guided by the Deputy for Meteorology (Regulation of the Meteorology, Climatology and Geophysics Agency of the Republic of Indonesia Number 6 of 2020 concerning the Organization and Work Procedures of the Meteorology, Climatology and Geophysics Agency, Meteorological Stations, Climatological).

One of the tasks and functions of the Meteorological Station is to provide meteorological information services, which include public and special information. Public information consists of routine information (weather forecasts) and early warnings (extreme weather). In contrast, special information includes weather data for aviation, weather information for insurance claims and other specific meteorological data as required by the user (Republic of Indonesia Government Regulation Number 11 of 2016 concerning Meteorology, Climatology). Meteorological services emerge as a new source of meteorological information to support adaptation and mitigation decisions made by both public and private sectors at global, regional and cross-ecosystem scales (Liu et al. 2021).

Lampung Province itself has unique tectonic conditions, being traversed by two major plates: the Indo-Australian Plate and the Eurasian Plate. Generally, Lampung Province has a tropical climate with diverse climate types, characterised by a monsoonal pattern, where there is a noticeable difference between the rainy and dry seasons, as well as a single peak season. The complex geographical conditions and varied climate types in Lampung provide the region with abundant natural resources but also make it vulnerable to natural hazards. The level of vulnerability to natural hazards in Lampung Province comprises 15 administrative districts, predominantly categorised as having moderate (12 administrative regions) and high vulnerability (3 administrative regions). Lampung Province not only faces the risk of geological disasters such as earthquakes, tsunamis, volcanic eruptions and landslides but also is vulnerable to various hydrometeorological disasters, including drought, heavy rainfall, forest fires, strong winds, tornadoes and floods (Manik et al. 2017; Sulistyawati, Zulkarnain & Halengkara 2021; Wibisono 2019).

The Class I Radin Inten II Meteorological Station has a major role in supporting the government by providing timely, precise and accurate weather information services. This service plays a crucial role in protecting the people of Lampung from potential dangers and losses, both in terms of material and life safety (Mills 2007). This station routinely provides the latest meteorological data, including weather forecasts and various information related to weather conditions (Robinson et al. 2018). Additionally, this station plays a vital role in providing early warnings about extreme weather to the public (Larsen & Skrumsager 2000).

The Meteorological Station plays a crucial role in maintaining flight safety by providing critical weather information that supports airport operations. This service includes emergency weather warnings and special guidance for take-off and landing processes (DeBlasio et al. 1996). To ensure flight safety, this station also provides meteorological data tailored to flight routes and operational needs (Kustiani & Siregar 2017). In addition, detailed weather briefings are provided to pilots and flight crews to support smooth operations (Hyman et al. 2006).

In addition to providing primary meteorological services, the station also prepares supporting documents for flights and actively participates in seminars, conferences and community service activities (Supadi 2022). To ensure efficient and targeted public services, the station has designed strategic planning as a framework for achieving the best service standards, including standard operating procedures (SOPs), for the community (Hoffman 2015; Kettl & Kelman 2007; Paron 2000). The scientific and social value of this research lies in its exploration of adaptive public management strategies within disaster management frameworks, specifically through the lens of mass public warning systems. By analysing the implementation of these systems in Lampung, this study aims to contribute valuable insights into how government agencies can optimise their disaster response strategies and improve public safety. This research is socially relevant in that it directly addresses the needs of communities living in disaster-prone areas, empowering them with timely and reliable information, a service the station is committed to providing. Scientifically, the study builds on the concept of adaptive governance, providing evidence-based recommendations to enhance the efficiency and inclusiveness of public warning systems.

The primary objective of this study is to evaluate the role of the Class I Meteorological Station Radin Inten II in adapting its management strategies to better respond to the growing challenges posed by natural hazards in Lampung. A key focus of the study is on the station’s capacity to collaborate across agencies, as this is crucial in disaster management. The study also aims to assess the station’s engagement with the local community and its ability to disseminate accurate and timely weather information. It aims to identify key factors that contribute to the success or limitations of the station’s efforts in implementing effective public warning systems, focusing on the elements of adaptive governance such as coordination, social capital building and community empowerment. In line with these objectives, this research will also investigate the gaps and challenges in disaster management coordination, capacity development and the integration of adaptive governance principles, proposing ways to strengthen these systems to improve public safety and resilience in Lampung Province.

## Research methods and design

### Research design

Recent research in academic journals indicates that adaptive public management approaches play a significant role in strengthening government institutions, particularly in frontier, outermost and disadvantaged regions. Adaptive public management approaches are increasingly being recognised as essential for strengthening government institutions, especially in frontier, outermost and disadvantaged regions. These approaches emphasise the need for flexible and context-driven resource allocation, particularly in areas facing unique developmental challenges. For instance, Greijn et al. (2015) emphasise the importance of capacity development in enhancing institutional effectiveness across various regions, demonstrating that adaptive governance is crucial for addressing local and regional needs. Similarly, Maulana, Decman and Durnik (2022) explore how digital transformation can foster collaborative governance in Indonesian local governments by improving administrative efficiency and responsiveness.

The integration of adaptive management in policy-making is essential for addressing environmental and climate-related challenges. Karo and Kattel (2014) discuss how policy capacity and adaptive governance support sustainable development in disadvantaged regions, helping governments better respond to environmental crises. Furthermore, Cedergren and Hassel (2024) examine how building organisational adaptive capacity is vital for public sector resilience, particularly in times of crisis, to maintain governance stability. Additionally, they emphasise the role of adaptive education systems, which integrate blended learning and agile management practices to enhance education quality in crisis-affected frontier, outermost and disadvantaged areas (Rismawati et al. 2025).

Adaptive management emphasises the state’s ability to adapt to changing governance dynamics, particularly in the context of natural resource management. This approach provides a flexible framework that can be implemented by both central and local governments (Armitage 2006). Through this technique, adaptive public management facilitates cross-sector collaboration and enhances the ability of social-ecological systems to respond to existing complexities (Abrams et al. 2020; Folke et al. 2005). This approach ensures that governance structures can keep up with evolving community needs and environmental changes by encouraging decentralised policies and adaptive responses (Emerson & Gerlak 2015; Huitema et al. 2009). To support sustainable resource management and policy effectiveness, adaptive governance also focuses on enhancing the capacity of local governments, which is an essential element in adaptive public management practices (Larson & Soto 2008; Rijke et al. 2012).

This study is based on the adaptive governance theory, which emphasises the government’s capacity to respond to challenges dynamically and provide guidance for governance at the local and national levels. This approach strengthens the function of various elements in the bureaucracy, regulations and managerial structures that play a role in decision-making and policy implementation.

### Methods

This study adopts a qualitative approach, aiming to gain an in-depth understanding of adaptive governance by analysing data through case studies and interviews. The study primarily aims to identify the key elements involved in implementing adaptive governance, particularly within the context of disaster management and mitigation. To this end, the study reviewed relevant literature and statistical data related to the use of mass public warning systems, providing a foundation for the analysis. Interviews were conducted with key stakeholders who play pivotal roles in disaster management, including government agencies, meteorological organisations and emergency response teams. The population for these interviews consisted of experts and personnel from agencies directly involved with disaster risk management. Specifically, the study focused on participants from the UPT of the Class I Radin Inten II Meteorological Station, under the Meteorology, Climatology and Geophysics Agency (Badan Metereologi, Klimatologi, and Geofisika [BMKG]). The participants were selected using purposive sampling, a method designed to target individuals with substantial experience and knowledge regarding the implementation and use of mass public warning systems in disaster scenarios.

A total of 15 participants were interviewed for this study. These interviews included meteorologists, disaster response officers, local government officials and other relevant stakeholders who have experience with the mass public warning systems during recent disaster situations. A semi-structured interview format was employed, allowing for flexibility to explore the participants’ experiences while ensuring that all interviewees addressed similar topics to maintain consistency. The data gathered from these interviews were triangulated with other data sources, such as case studies, document analysis and direct observation, which were focused on the use of mass public warning systems during recent disasters. Alongside the interviews, document analysis was conducted to collect and review relevant policies, reports and official documents concerning the mass public warning systems. Direct observation of disaster response operations was also part of the study, providing an additional layer of contextual insight into the interview findings. This multi-method approach ensured a comprehensive analysis of adaptive governance in disaster management, with a particular focus on the role of the mass public warning system.

### Ethical considerations

Ethical approval to conduct this study was obtained from the University of Lampung Social and Political Science Faculty Ethics Committee (No. 52/UN.26.16/PN.02.00.01/2024).

## Results and discussion

### Overview of Radin Inten II class I meteorological station

Radin Inten II Class I Meteorological Station, based in Lampung, is one of the regional UPTs under the umbrella of the Meteorology, Climatology and Geophysics Agency. In terms of administration, its duties and functions are overseen by the Principal Secretary, while technical and operational oversight falls under the Deputy for Meteorology. However, the station ultimately remains under the responsibility of the Head of Meteorology, Climatology and Geophysics Agency. As the Agency regional representative, Radin Inten II Station carries out critical tasks, such as meteorological observation, data management, service delivery, meteorological services and equipment maintenance, by Regulation of the Meteorology, Climatology and Geophysics Agency of the Republic of Indonesia Number 6 of 2020 concerning the Organization and Work Procedures of the Meteorology, Climatology and Geophysics Agency, Meteorological Stations, Climatological.

**FIGURE 1:**
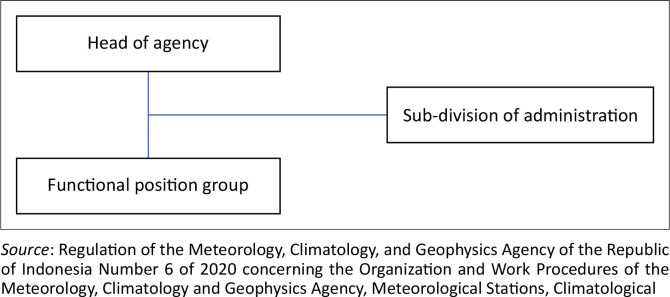
Organizational structure of Radin Inten II class I meteorological station.

Meteorological Station Radin Inten II is led by a Station Head who oversees the Subdivision of General Affairs and the Functional Position Group (see Figure 1). The General Affairs Subdivision handles everything from office administration, HR and finance to housekeeping, work programmes and reporting. Meanwhile, the Functional Position Group takes care of all the technical services, aligning their expertise with their tasks. Their roles are created based on station needs, and their appointment follows existing regulations.

This research focuses on key indicators such as coordination, timely and accurate information management and stakeholder engagement. The primary objective was to identify factors that contribute to success or present challenges in the development of an inclusive early warning system. Through this case study, the analysis examines the implementation of adaptive governance in enhancing the effectiveness of public warning systems by promoting public participation, strengthening inter-agency coordination, ensuring policy flexibility and responding efficiently to emergencies. The goal is to ensure that the system is adaptive, responsive and capable of delivering critical information when it is most needed.

### Collaboration

According to Sharma, Bhadwal and Singh (2018), cross-actor collaboration within adaptive governance necessitates the integration of both formal and informal cooperation to achieve inclusivity. However, in the case of the Meteorology, Climatology and Geophysics Agency in Lampung, inter-agency collaboration in disaster management remains suboptimal, often restricted to sporadic coordination with the Regional Disaster Management Agency and national media outlets such as Indonesian National Television (*TVRI*) and Indonesian National Radio (*RRI*) for information dissemination (Rahmawan et al. 2024). Media-based communication systems have shown effectiveness, as evidenced in Southeast Sulawesi, where the Meteorology, Climatology and Geophysics Agency and the Regional Disaster Management Agency collaborated to disseminate disaster-related weather information and early warnings, supported by adaptive public management practices (Sirajuddin et al. 2022). The use of information and communication technology (ICT) also strengthens public awareness of hydrometeorological disasters through such partnerships (Ruslanjari et al. 2023). Similar collaborations, such as those in Aceh and Gunungkidul, demonstrate the significance of partnerships between the Meteorology, Climatology and Geophysics Agency and the Regional Disaster Management Agency in managing disaster risks and ensuring accurate information dissemination (Nurhadi 2014).

### Community empowerment and engagement

Community involvement plays a crucial role in adaptive governance, especially at all stages of policy implementation, from planning to evaluation. For instance, early engagement in disaster mitigation and climate adaptation policies proves key to programme effectiveness, as highlighted by Ray and Wray (2024), who underscores the importance of active community participation, particularly among young people, in strengthening disaster risk management and climate adaptation strategies.

**FIGURE 2:**
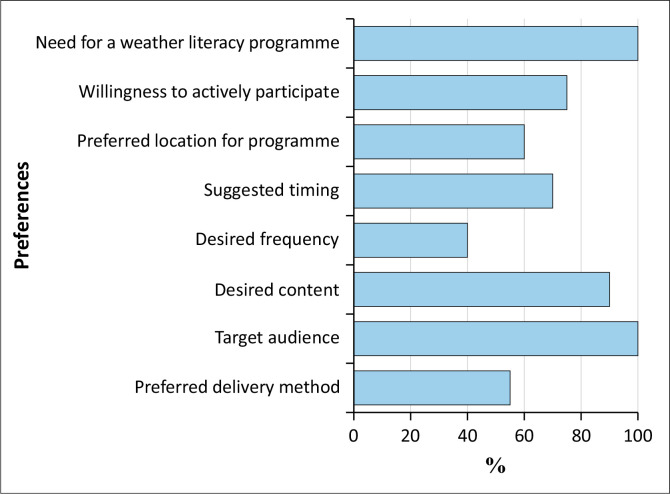
Community preferences for weather literacy programme.

In Lampung Province, the Meteorology, Climatology and Geophysics Agency has utilised digital platforms to disseminate disaster risk information. However, survey data from affected areas in Bandar Lampung indicate that the community desires more than merely receiving information. The questionnaire findings reveal that 100% of respondents agree on the need for weather literacy programmes, with 75% expressing willingness to actively participate. Furthermore, slightly more than half (60%) suggest that such programmes be held at local village or subdistrict offices, with a preference for scheduling on weekends (70%), reflecting a demand for accessible and convenient timing. These insights highlight a significant potential for greater community engagement in disaster risk management processes (see Figure 2).

These findings support the argument by Hajar et al. (2024) that direct community engagement can enhance the effectiveness of local interventions, with adaptive public management more effectively meeting local needs through community inclusion. Additionally, Swaris, Halwatura and Amaratunga (2024) emphasise the importance of consistency, diversity and trust in interactions between government and communities, so that climate change adaptation, disaster risk reduction and sustainable development goals are achieved. Survey data show that slightly more than half of residents prefer programme delivery through seminars or dissemination (55%), highlighting a need for a more interactive and educational approach.

Further studies underscore the significance of community-based initiatives in enhancing resilience, as seen in Bangladesh, where community-led projects have bolstered local resilience (Islam, Ahmad & Khan 2024). Likewise, Jain et al. (2024) illustrate how community-based water management systems in India have contributed significantly to climate adaptation efforts. In the context of Lampung, trust in the community’s ability to engage in disaster mitigation remains limited although the survey data analysis reflects strong potential for sustained engagement. The efficacy of more inclusive mitigation initiatives can be strengthened, and community trust can be raised by increasing direct participation in catastrophe planning and response.

### Capacity development

One important aspect of adaptive governance is capacity building, the success of which is highly dependent on the quality of solid leadership. Leaders have a key role in redefining the challenges faced, building partnerships between stakeholders and managing the distribution of resources to ensure the sustainability of governance initiatives. Atilano-Tang (2023) stated that leadership is very important in encouraging active participation from stakeholders, which is key to the sustainability of governance projects. Leaders, as the main element in adaptive governance, are responsible for encouraging community involvement and implementing innovative managerial strategies. For an organisation to function optimally, leadership development at all levels is essential. According to Díez-Vial and Belso-Martinez (2024), leadership development plays an important role in improving an organisation’s ability to adapt, especially in the context of disaster risk management. In Lampung Province, the Meteorology, Climatology and Geophysics Agency implemented a structured approach to leadership development by systematically collecting feedback through surveys and evaluations to identify areas for improvement. As part of this effort, managerial training specifically designed to address capacity gaps at various levels of the organisation was also implemented. Effective leadership promotes emergency readiness in addition to team cooperation. According to Niamir et al. (2024), leadership is essential for strengthening climate resilience through planned actions, particularly in urban settings. The Meteorology, Climatology and Geophysics Agency in Lampung Province applies these principles by conducting daily assemblies to enhance coordination and ensure readiness for disaster response.

Collaboration with institutions such as the National Research and Innovation Agency and the National Institute of Public Administration further strengthens leadership development by integrating contemporary innovations. Ramani-Chander et al. (2024) assert that effective leadership is essential for managing diverse stakeholder groups and scaling interventions, which is crucial for achieving long-term governance success. This comprehensive approach to leadership development, characterised by collaboration and continuous improvement, reinforces the adaptive capacity of governance frameworks in the face of evolving challenges.

### Knowledge and decision-making

The provision of accurate and timely information is crucial in adaptive governance, as it enables stakeholders to analyse, evaluate and make informed decisions. In the realm of disaster risk management, early warning systems play a vital role in reducing risks. Trogrlić et al. (2022) emphasise the significance of coordinated information dissemination for effective emergency response. Within the Meteorology, Climatology and Geophysics Agency in Lampung Province, the ‘Knowledge and Decision-making’ indicator ensures that weather, climate and disaster warnings are based on verified data. The use of technologies such as WhatsApp expedites the communication of this information, facilitating a swift response to emergencies.

Despite the efforts made by the Meteorology, Climatology and Geophysics Agency, challenges persist in coordinating with external agencies, as there is a tendency to prioritise information dissemination over active collaboration. Twigg (2021) highlights that the sustainability of early warning systems relies not only on effective information dissemination but also on robust inter-agency collaboration. Seng (2012) stresses that enhancing governance frameworks and fostering inter-sectoral partnerships are essential for improving disaster preparedness and response. Additionally, Pulwarty and Sivakumar (2018) argue that reliable information systems are crucial in early warning contexts, particularly for managing risks such as droughts. Strengthening cross-agency collaboration and improving information systems will contribute to more comprehensive disaster mitigation efforts, ensuring that governance frameworks are responsive to the complexities of disaster management.

### Identified gap

The Meteorology, Climatology and Geophysics Agency plays a crucial role in adaptive governance, particularly in disaster management. Olhoff (2011) highlights the need for integrating climate adaptation and disaster risk reduction through strong inter-agency collaboration and decision-making. The agency has advanced capacity-building efforts with structured training and accurate dissemination of weather and disaster information. However, challenges remain, especially in external collaboration. Bisri and Lutfiananda (2022) point out that ASEAN countries, including Indonesia, face difficulties in inter-agency cooperation, which limits effective disaster responses. The Meteorology, Climatology and Geophysics Agency’s dependence on internal messaging apps like Zoom Embryo and WhatsApp highlights the absence of proactive external cooperation.

In addition, community engagement by these institutions is limited because of the lack of a clear coordination framework. Uchiyama, Ismail and Stevenson (2021) argue that an approach that involves communities and better governance is essential in disaster risk management. The Meteorology, Climatology and Geophysics Agency needs to develop a more inclusive relationship with local government communities to strengthen disaster preparedness. Leadership also plays a crucial role. In the context of Indonesia’s adaptation to climate change, Yoseph-Paulus and Hindmarsh (2018) highlight the importance of leadership in strengthening inter-sectoral collaboration and increasing capacity. Increasing the capacity of institutions in managing disaster risk can be achieved by strengthening leadership and increasing inter-agency cooperation.

## Conclusion

The success of the Class I Radin Inten II Meteorological Station within the framework of adaptive governance depends on various mutually supportive elements, such as collaboration, coordination, community participation, capacity building and timely information distribution. The Meteorology, Climatology and Geophysics Agency in Lampung Province is responsible for important tasks, such as meteorological observations and data management, which ultimately contribute to the effective delivery of meteorological services. In addition, it involves stakeholder involvement and ensures the effective dissemination of information that supports disaster management.

However, effective disaster management requires cooperation with the Regional Disaster Management Agency, and inter-agency interaction is currently inadequate, frequently restricted to sporadic coordination. While awareness of hydrometeorological disasters has been raised by efficient communication systems backed by flexible public management techniques, the absence of formal standard and passive inter-agency contact continues to hinder the overall effectiveness of disaster response. Therefore, this study recommends the development of structured SOPs for disaster management and enhanced inter-agency collaboration to ensure a more coordinated, timely and effective response during disasters.

Digital communication initiatives have shown promise in promoting stakeholder engagement and building resilience, but broader community involvement in decision-making processes remains limited. This research highlights that community empowerment and engagement are critical for improving disaster management strategies. Active participation from communities enhances the effectiveness of local interventions and ensures that disaster mitigation efforts align with the needs of the population. Therefore, this study recommends implementing inclusive community-based programmes that actively involve local residents in disaster preparedness and mitigation planning. These initiatives should be designed to build trust, raise awareness and foster a sense of shared responsibility among community members.

The study also emphasises the importance of capacity development through effective leadership. Leadership is a key element in fostering adaptive governance, and this research underscores the need for a systematic approach to leadership development that addresses existing capacity gaps within the Meteorology, Climatology and Geophysics Agency. The study recommends the establishment of leadership training programmes that focus on strengthening decision-making and coordination skills, particularly for managing disaster response in dynamic and complex environments.

Furthermore, there are several recommendations for disaster management strategy development in the future:

- Development of structured SOPs for disaster management to ensure more consistent and effective inter-agency collaboration.
- Enhanced inter-agency collaboration to improve coordination and response times during disasters, especially between the Meteorology, Climatology and Geophysics Agency, the Regional Disaster Management Agency and local communities.
- Implement inclusive community-based programmes to engage residents in disaster preparedness and response efforts.
- Leadership training programmes for improving decision-making and coordination during crises, particularly focusing on adaptive governance and disaster management.
- Strengthening the early warning systems by improving collaboration and information dissemination channels to ensure timely and accurate responses to emerging disaster threats.

Finally, providing accurate and timely information is essential for informed decision-making in disaster contexts. Early warning systems mitigate risks, but their sustainability depends on not just the accuracy of the data but also on robust inter-agency collaboration and efficient dissemination mechanisms. Strengthening these systems through the integration of structured SOPs, regular coordination meetings and more dynamic engagement with local communities will be crucial for enhancing the overall effectiveness of disaster management strategies in Lampung Province.
